# Supplementary material for: Exposure to the Amino Acids Histidine, Lysine, and Threonine Reduces mTOR Activity and Affects Neurodevelopment in a Human Cerebral Organoid Model
Source: Nutrients. 2022 May 23;14(10):2175. doi: 10.3390/nu14102175 (PMC9145399; doi:10.3390/nu14102175)
Supplement: Supplementary file 1 [file nutrients-14-02175-s001.zip › Supplementary figures.pptx]

## Slide 1
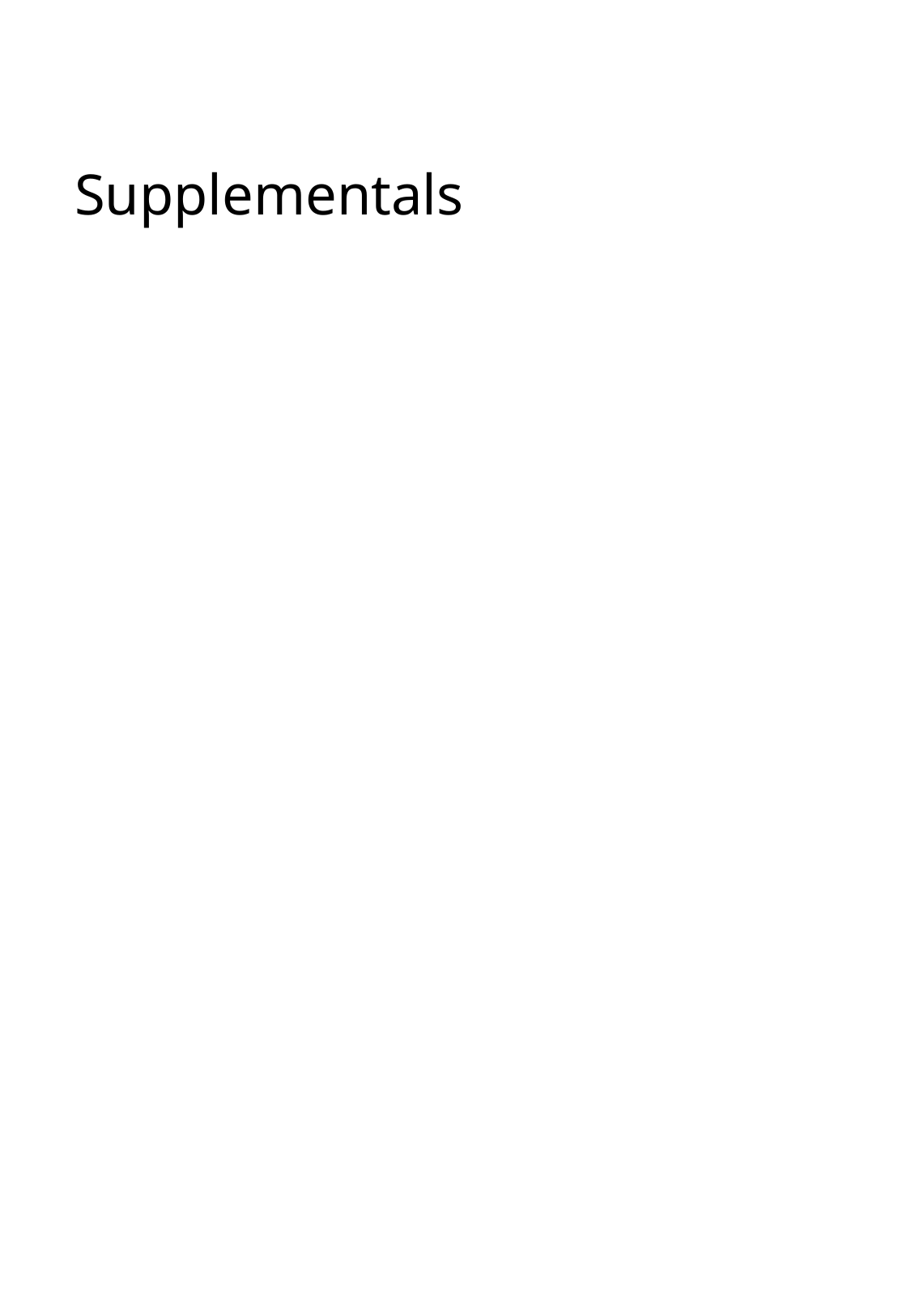

# Supplementals

## Slide 2
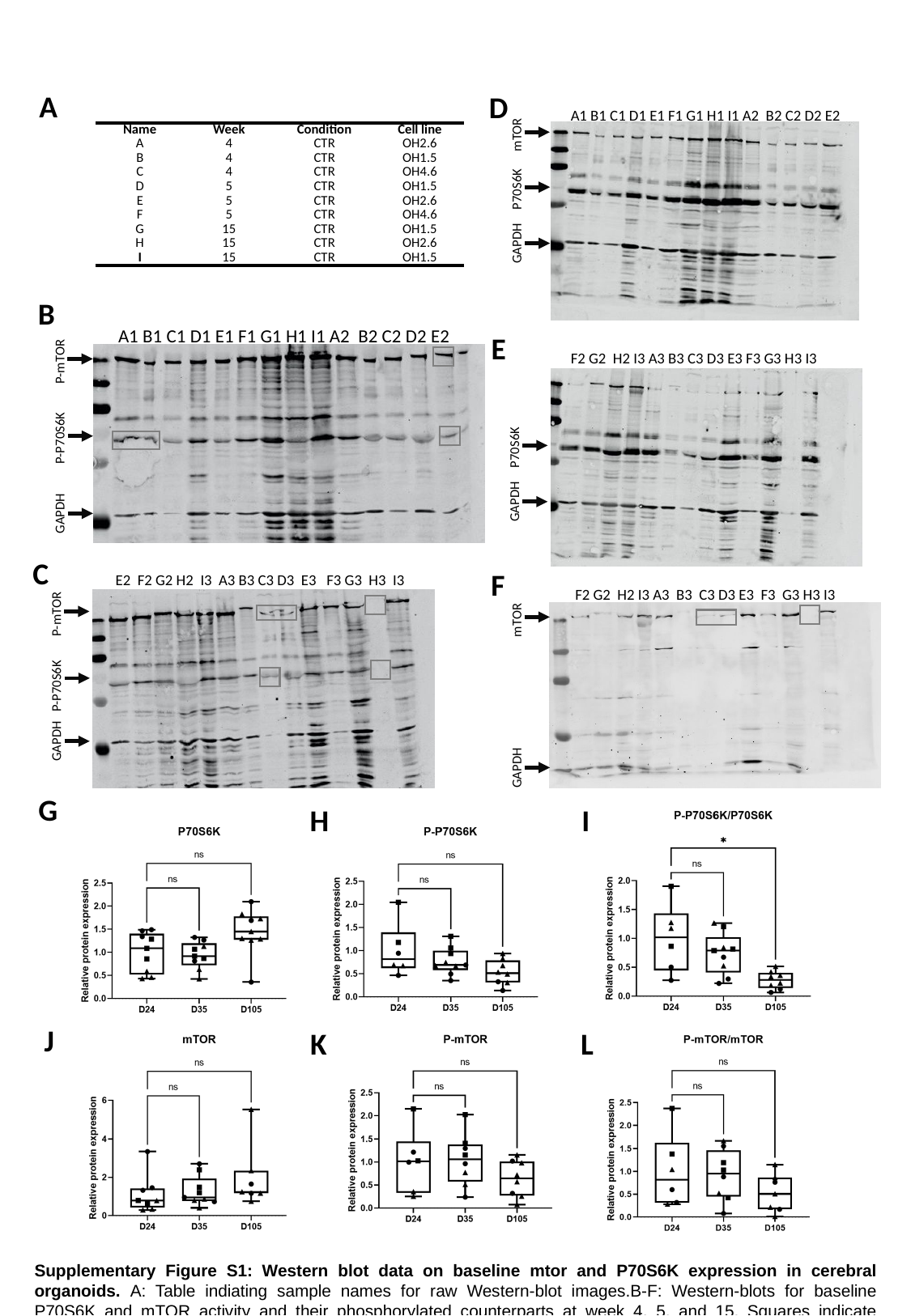

A
D
A1 B1 C1 D1 E1 F1 G1 H1 I1 A2 B2 C2 D2 E2
mTOR
| Name | Week | Condition | Cell line |
| --- | --- | --- | --- |
| A | 4 | CTR | OH2.6 |
| B | 4 | CTR | OH1.5 |
| C | 4 | CTR | OH4.6 |
| D | 5 | CTR | OH1.5 |
| E | 5 | CTR | OH2.6 |
| F | 5 | CTR | OH4.6 |
| G | 15 | CTR | OH1.5 |
| H | 15 | CTR | OH2.6 |
| I | 15 | CTR | OH1.5 |
P70S6K
GAPDH
B
A1 B1 C1 D1 E1 F1 G1 H1 I1 A2 B2 C2 D2 E2
E
F2 G2 H2 I3 A3 B3 C3 D3 E3 F3 G3 H3 I3
P-mTOR
P-P70S6K
P70S6K
GAPDH
GAPDH
C
F
E2 F2 G2 H2 I3 A3 B3 C3 D3 E3 F3 G3 H3 I3
F2 G2 H2 I3 A3 B3 C3 D3 E3 F3 G3 H3 I3
P-mTOR
mTOR
P-P70S6K
GAPDH
GAPDH
G
I
H
J
K
L
Supplementary Figure S1: Western blot data on baseline mtor and P70S6K expression in cerebral organoids. A: Table indiating sample names for raw Western-blot images.B-F: Western-blots for baseline P70S6K and mTOR activity and their phosphorylated counterparts at week 4, 5, and 15. Squares indicate excluded samples for technical exclusion criteria. G-L: Quantification of western-blots for mTOR, phospho-mTOR and the P-mTOR/mTOR ratio, and P70S6K, phospho-P70S6K and the P-P70S6K/P70S6K ratio in CTR organoids at different timepoints (week 4, 5, and 15). Boxplots display median and IQR, with whiskers from minimum to maximum value. Data points represent individual organoids from different cell-lines (circles = OH2.6; triangles = OH1.5; squares = OH4.6). Kruskall-Wallis tests with Dunn test for multiple comparisons was used to compare different timepoints (* p < 0.05; ** p < 0.01).

## Slide 3
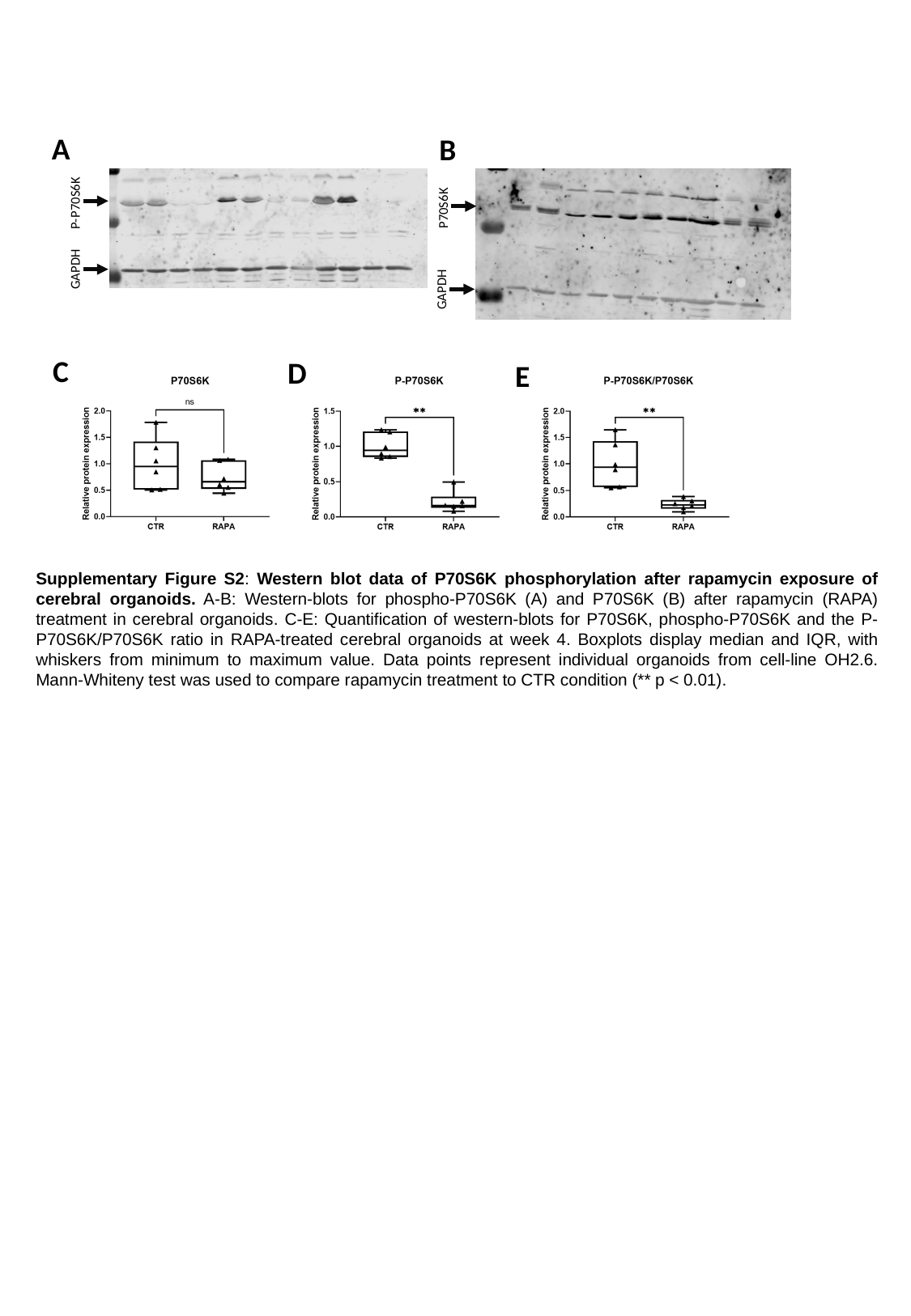

A
B
P70S6K
P-P70S6K
GAPDH
GAPDH
C
D
E
Supplementary Figure S2: Western blot data of P70S6K phosphorylation after rapamycin exposure of cerebral organoids. A-B: Western-blots for phospho-P70S6K (A) and P70S6K (B) after rapamycin (RAPA) treatment in cerebral organoids. C-E: Quantification of western-blots for P70S6K, phospho-P70S6K and the P-P70S6K/P70S6K ratio in RAPA-treated cerebral organoids at week 4. Boxplots display median and IQR, with whiskers from minimum to maximum value. Data points represent individual organoids from cell-line OH2.6. Mann-Whiteny test was used to compare rapamycin treatment to CTR condition (** p < 0.01).

## Slide 4
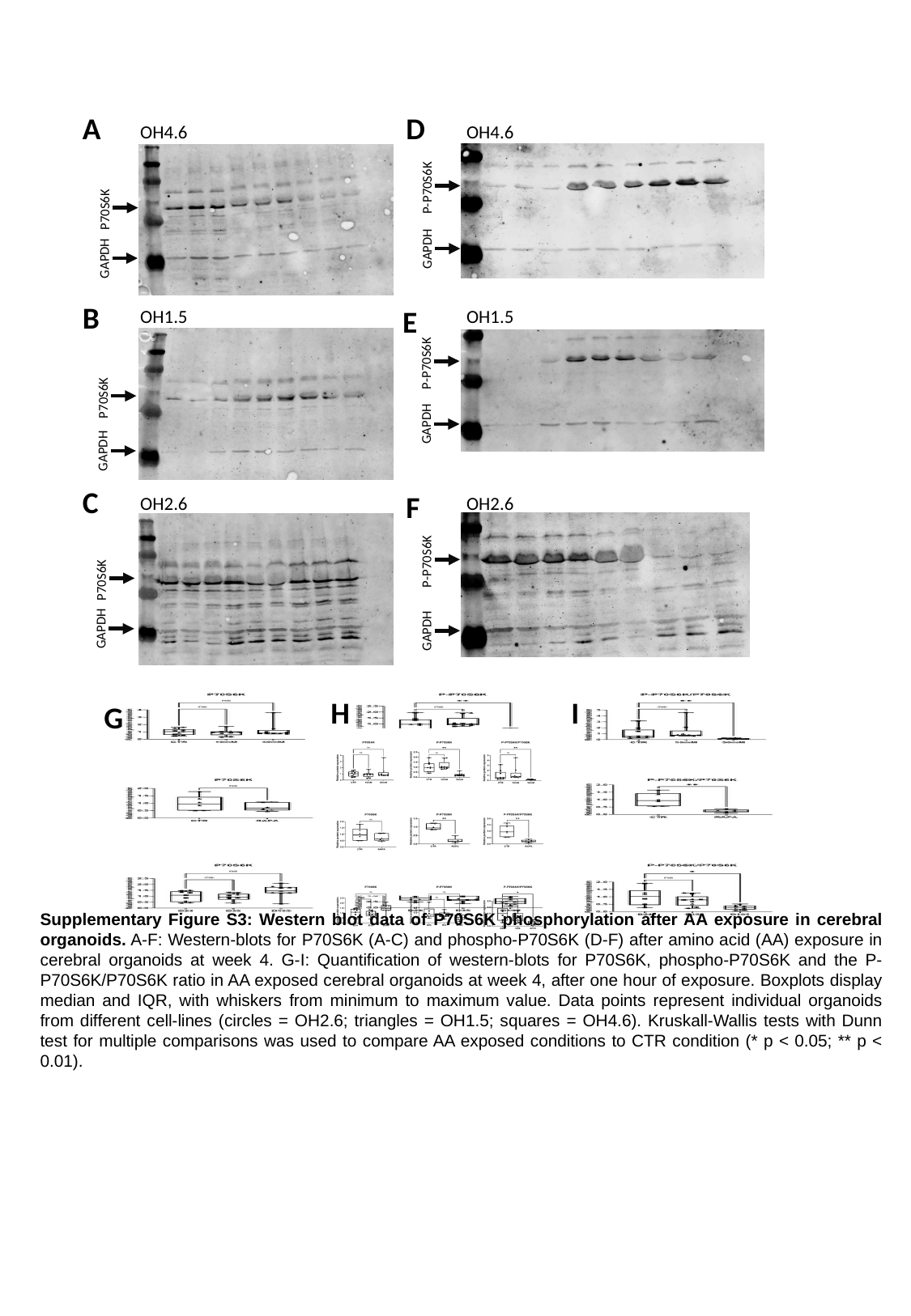

A
D
OH4.6
OH4.6
P-P70S6K
P70S6K
GAPDH
GAPDH
OH1.5
OH1.5
P-P70S6K
P70S6K
GAPDH
GAPDH
OH2.6
OH2.6
P-P70S6K
P70S6K
GAPDH
GAPDH
B
E
C
F
I
H
G
Supplementary Figure S3: Western blot data of P70S6K phosphorylation after AA exposure in cerebral organoids. A-F: Western-blots for P70S6K (A-C) and phospho-P70S6K (D-F) after amino acid (AA) exposure in cerebral organoids at week 4. G-I: Quantification of western-blots for P70S6K, phospho-P70S6K and the P-P70S6K/P70S6K ratio in AA exposed cerebral organoids at week 4, after one hour of exposure. Boxplots display median and IQR, with whiskers from minimum to maximum value. Data points represent individual organoids from different cell-lines (circles = OH2.6; triangles = OH1.5; squares = OH4.6). Kruskall-Wallis tests with Dunn test for multiple comparisons was used to compare AA exposed conditions to CTR condition (* p < 0.05; ** p < 0.01).

## Slide 5
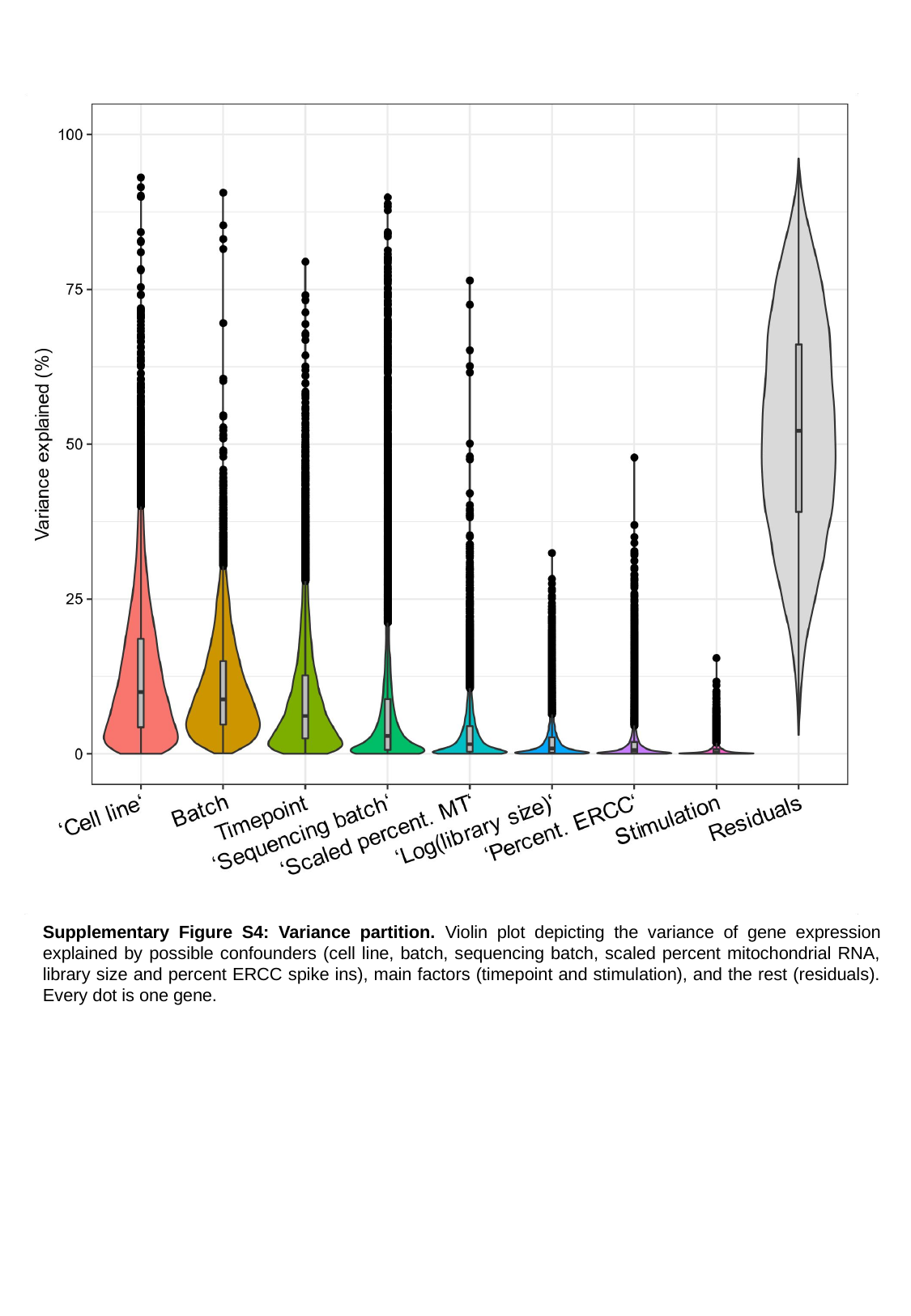

Supplementary Figure S4: Variance partition. Violin plot depicting the variance of gene expression explained by possible confounders (cell line, batch, sequencing batch, scaled percent mitochondrial RNA, library size and percent ERCC spike ins), main factors (timepoint and stimulation), and the rest (residuals). Every dot is one gene.

## Slide 6
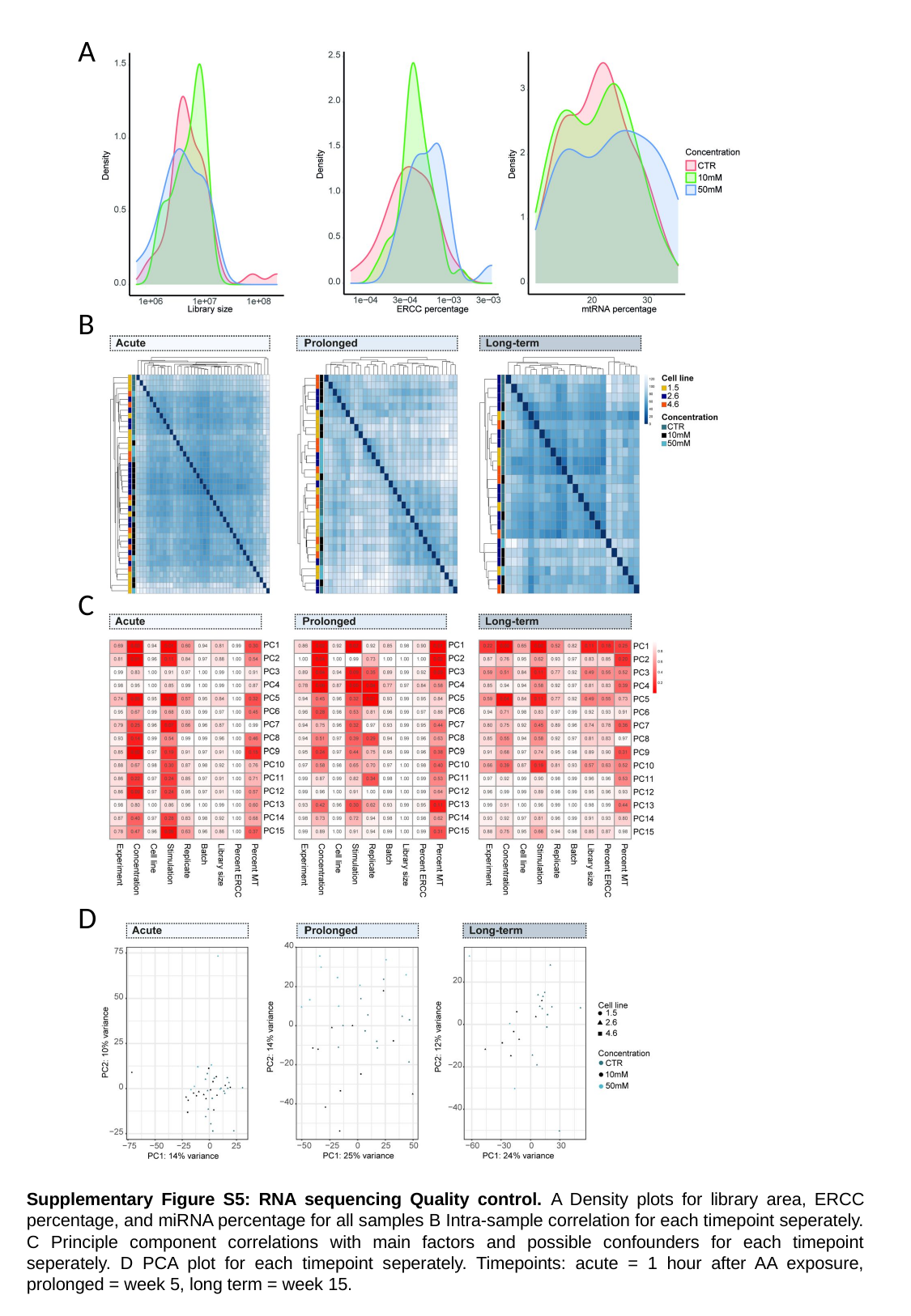

A
B
C
D
Supplementary Figure S5: RNA sequencing Quality control. A Density plots for library area, ERCC percentage, and miRNA percentage for all samples B Intra-sample correlation for each timepoint seperately. C Principle component correlations with main factors and possible confounders for each timepoint seperately. D PCA plot for each timepoint seperately. Timepoints: acute = 1 hour after AA exposure, prolonged = week 5, long term = week 15.

## Slide 7
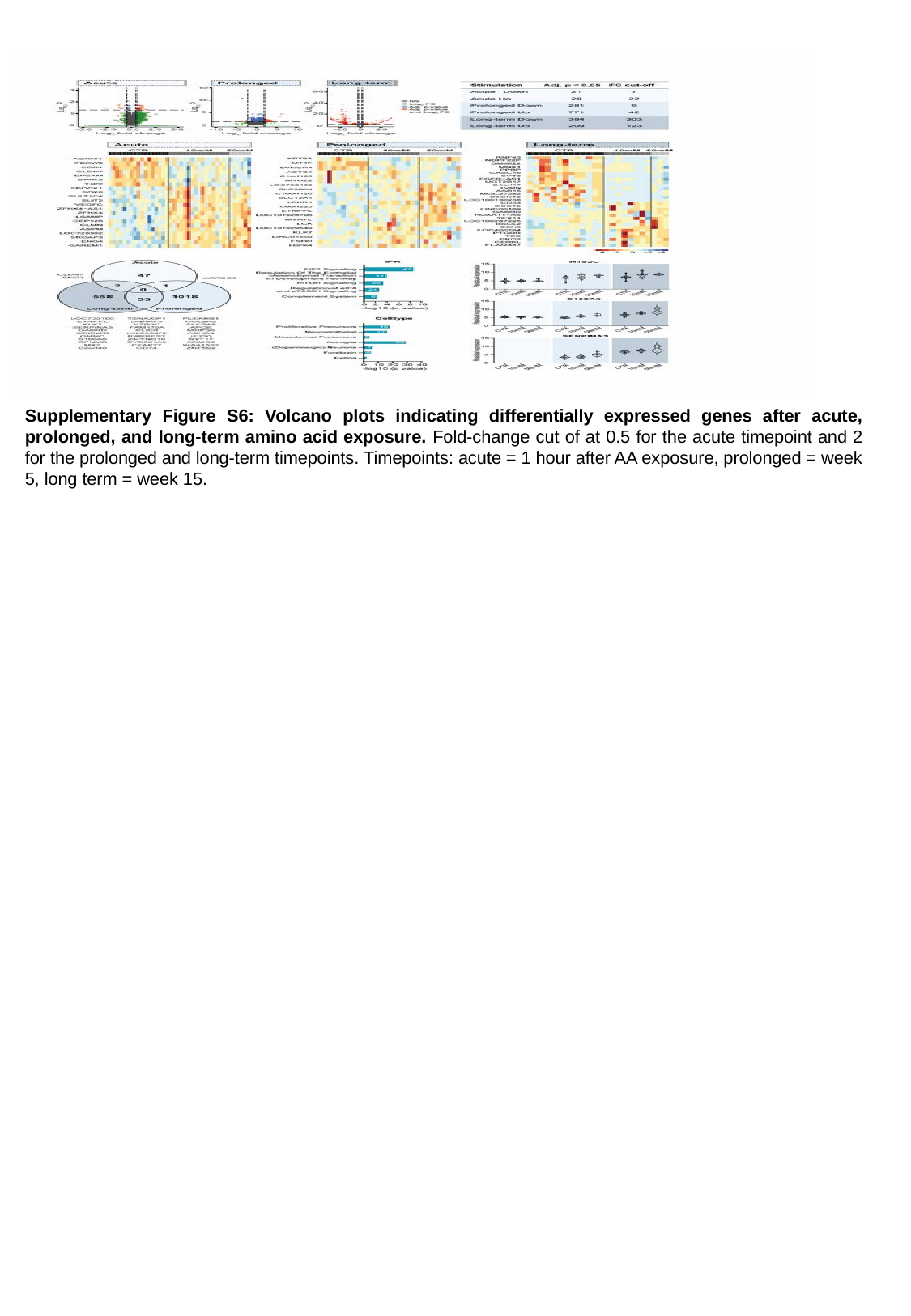

Supplementary Figure S6: Volcano plots indicating differentially expressed genes after acute, prolonged, and long-term amino acid exposure. Fold-change cut of at 0.5 for the acute timepoint and 2 for the prolonged and long-term timepoints. Timepoints: acute = 1 hour after AA exposure, prolonged = week 5, long term = week 15.

## Slide 8
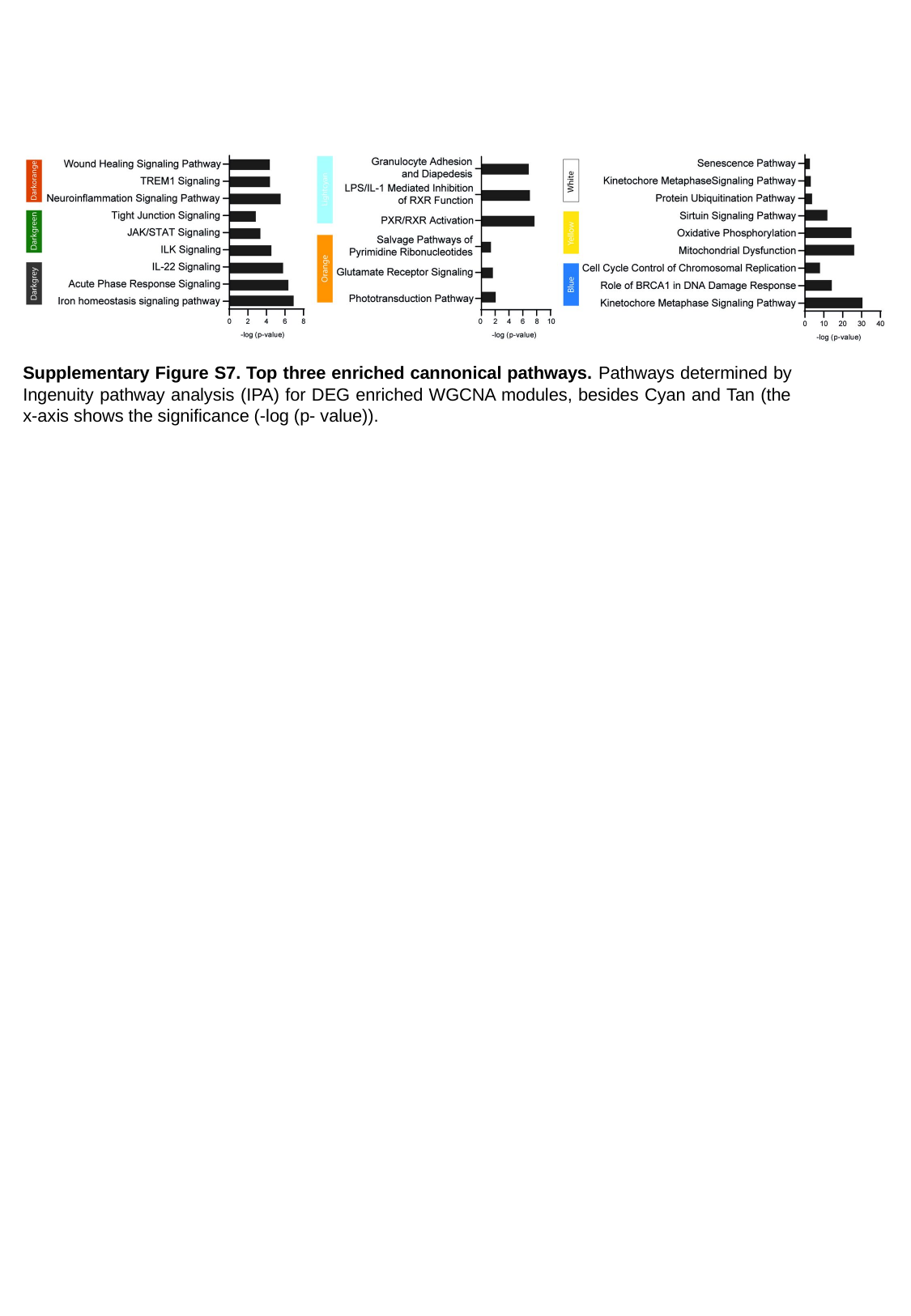

Supplementary Figure S7. Top three enriched cannonical pathways. Pathways determined by Ingenuity pathway analysis (IPA) for DEG enriched WGCNA modules, besides Cyan and Tan (the x-axis shows the significance (-log (p- value)).
